# Life satisfaction and mental health from age 17 to 21 years in a general population sample

**DOI:** 10.1007/s12144-022-03685-9

**Published:** 2022-10-07

**Authors:** Jose Marquez, Ioannis Katsantonis, Ruth Sellers, Gundi Knies

**Affiliations:** 1 Manchester Institute of Education, University of Manchester, Oxford Road, M13 9PL Manchester, UK; 2 Psychology, Education and Learning Studies Research Group, Faculty of Education, University of Cambridge, 184 Hills Rd, CB2 8PQ Cambridge, UK; 3 Brighton & Sussex Medical School, University of Sussex, Falmer, UK; 4 Faculty of Education, University of Cambridge, Cambridge, UK; 5 Johann Heinrich von Thünen-Institut, Institute of Rural Studies, Bundesallee 64, 38116 Braunschweig, Germany

**Keywords:** Mental health, Wellbeing, Life satisfaction, Adolescence, Young adults, Gender, Direction of effects

## Abstract

Adolescence is a period when both mental health (MH) and wellbeing start deteriorating, which raises the question of how the two phenomena are linked and whether deterioration in one might be used to flag problematic developments in the other. While research shows that wellbeing and MH are associated, the direction of the association is not clear and longitudinal analyses, that might help disentangle the cause and effect, are scarce. Moreover, few studies have investigated the directional relation between MH and wellbeing early in the life course. In emerging adulthood, evidence indicates reciprocal associations and no gender differences, whereas, in early and middle adolescence, results are mixed and differ across gender. Thus, we investigated the relationship between MH and wellbeing and the moderating effect of gender in the crucial developmental transition from middle adolescence to emerging adulthood. We undertake a cross-lagged longitudinal data analysis from a pooled sample of six pseudo-cohorts, including information from 661 young people who participated in the UK Household Longitudinal Study at ages 17, 19, and 21. Using a 7-points overall life satisfaction (LS) scale as an index of wellbeing and the 12-item General Health Questionnaire as a measure of MH, we found no associations between LS and MH in the 17–19 transition and bidirectional associations in the 19–21 transition. There were no substantial gender differences in either transition. We conclude that LS and MH predict each other in the transition from late adolescence (age 19) to emerging adulthood (age 21) for both males and females.

## Introduction

Research has shown that adolescence and emerging adulthood are crucial periods for wellbeing and mental health (MH), since this is a stage with many changes in relationships and social roles and the adoption of responsibilities (Arnett, 2007; Tanner & Arnett, 2016). Many MH symptoms may onset in adolescence, with a higher prevalence of depression and anxiety in females and a higher prevalence of substance use syndromes and suicide in males (Blakemore, 2019; Collishaw, 2015; Kessler & Zhao, 1999). Similarly, research has shown that wellbeing (mainly life satisfaction, LS) declines in adolescence and emerging adulthood (e.g., Blanchflower, 2021; Chen et al., 2020; Goldbeck et al., 2007; Knies, 2021; Marquez & Long, 2021), with lower starting values and more marked declines in females rather than males (Chui & Wong, 2016; Goldbeck et al., 2007; Herke et al., 2019; Kaye-Tzadok et al., 2017; Knies, 2021). However, there is still limited understanding of the longitudinal associations between these two constructs, particularly across adolescence and in the transition to emerging adulthood. To address this gap in extant research, we examined the developmental relationship between MH and LS during this critical transition.

### What do we mean by wellbeing and mental health?

Wellbeing has been conceptualized as involving both eudaimonic (psychological) and hedonic (subjective) wellbeing. Psychological wellbeing is often defined in terms of self-actualization and having a meaningful purpose in one’s life. Subjective wellbeing has been conceptualized to include an affective component (experiencing positive and negative affect, moods and feelings) and a cognitive element (satisfaction with different aspects of life and with life as a whole, namely overall LS) (Diener et al., 2002, Rees et al., 2013). Admittedly, there is less consensus on the definition of eudaimonic wellbeing compared to hedonic wellbeing (Katsantonis, 2022; Vik & Carlquist, 2018), and most existing research explored the developmental relationship between wellbeing and MH focusing on LS (Lyon et al., 2014; Fergusson et al., 2015; Patalay & Fitzsimons, 2018; Bieda et al., 2019). Therefore, in this study, we are focusing on LS. At the same time, in empirical studies examining both WB and MH, the latter tends to be defined in terms of the presence of mental ill-health symptoms of internalizing difficulties (anxiety, social anxiety, depression, somatic complaints, etc.), whereas the focus on externalizing difficulties (under-controlled, impulsive, or aggressive behaviour) is less common (Otto et al., 2021; Petersen et al., 2018). For this reason, but also due to limitations imposed by data availability, we operationalize MH in terms of internalizing difficulties. More details on how LS and MH are operationalized in this study are presented in the Methods section.

### Why study the association between wellbeing (life satisfaction) and mental health?

Historically wellbeing and MH have been considered opposite ends of a continuum. However, some theoretical frameworks, such as the complete state model of MH (Keyes, 2005), conceptualize wellbeing and MH as two correlated but separate dimensions, underscoring, thus, that the absence of symptoms of mental illness does not necessarily equate to high wellbeing –and reporting low wellbeing does not imply the presence of symptoms of poor MH. In line with this, there is increasing empirical evidence that these two psychological constructs form two domains/constructs that are associated but may have different correlates and may follow different trajectories over the life course (Patalay & Fitzsimons, 2016, 2018; Westerhof & Keyes, 2010, Sharp et al., 2016, Kinderman et al., 2015). The complete state model of MH has also inspired research looking into wellbeing as a mediator of MH and mental illness (Venning et al., 2011) and potential applications in fields such as trauma-informed positive education (Brunzell et al., 2016) and recovery (Slade, 2010). Therefore, there is empirical and theoretical evidence that underscores the importance of not treating wellbeing and MH as the same construct, and it is also clear that this has important implications in the field of MH as wellbeing promotion might be a promising route for reducing symptoms of poor MH. For this reason, it is important to advance our understanding of how wellbeing and MH may predict each other, especially in some critical developmental transitions characterized by increased vulnerability in these outcomes.

From a practical point of view, it is important to understand the directional nature of the relation between MH and wellbeing (life satisfaction) given the epidemiological trends indicating increments in adolescents’ internalizing and externalizing MH problems (Collishaw, 2015; Pitchforth et al., 2019), and the decrements in wellbeing (life satisfaction) (Thapar et al., 2021). Thus, it is necessary to gain in depth understanding of whether secular increases in MH may be driving decrements in wellbeing, or vice versa. This would help clinical and non-clinical professionals to design better interventions.

### What is the relationship between wellbeing and mental health in children and young people?

Evidence of associations between wellbeing and MH comes from two main sources. First, evidence from neuroscientific research shows that the neural correlates of wellbeing are many and complicated; nevertheless, some of the neural correlates of wellbeing are also implicated in the most common mental illnesses that are frequently partially defined as the absence of wellbeing (King, 2019). For example, areas of the prefrontal cortex (PFC) have been associated not only with wellbeing (Luo et al., 2014; Takeuchi et al., 2014) but also with symptoms of major depressive disorder (Keedwell et al., 2005; Rive et al., 2013) and anxiety (Gold et al., 2020; Spampinato et al., 2009). Another example is the correlation between the subcortical area of the thalamus with both wellbeing (King, 2019) and social anxiety (Brühl et al., 2011). Given the overlap between the neural correlates of wellbeing and MH, we expect that wellbeing will be reversely reciprocally related to MH.

The second source of evidence indicating a relationship between wellbeing and MH comes from cross-sectional and longitudinal analyses of survey data. Cross-sectional survey studies in children and young people show that LS, a central component of wellbeing, is negatively associated with psychosomatic symptoms (Piko, 2006), suicide risk (Zhang et al., 2014; Schapir et al., 2016), depression and anxiety (Dooley et al., 2015; Kelishadi et al., 2018; Zhang et al., 2014), school-related anxiety (Steinmayr et al., 2018; Marquez & Main, 2020), and substance abuse (Zullig et al., 2001). However, as these studies are cross-sectional, the temporal ordering and, thus, the direction of effects cannot be established.

Few studies have investigated the longitudinal associations between MH and wellbeing in children and young people, and most study this association unidirectionally. For example, previous research has found behavioural and emotional problems in childhood to predict lower LS in middle and late adolescence (Kjeldsen et al., 2016; Honkanen et al., 2011). Similarly, anxiety –specifically, school-related anxiety- has been found to predict adolescents’ LS and affective subjective wellbeing a year later (Steinmayr et al., 2016). Associations have also been found in research that focuses on the transition to adulthood. For example, Essau et al.(2014) found that anxiety in adolescence (age 14–19) predicted LS and other psychosocial outcomes at age 30, an association that was moderated by anxiety, substance- and alcohol-use disorder in emerging adulthood (age 24). Wang and Haworth (2020) found that childhood internalizing and externalizing problems were negatively associated with LS at age 23. However, neither study explicitly examined the direction of association between MH and wellbeing over time.

Only a few studies have investigated the direction of the effects between LS and MH problems in children and young people. Some have focused on early adolescence. For instance, in a sample of British adolescents, Patalay & Fitzsimons (2018) found age 11 LS to predict depressive symptoms at age 14 among males and females, and age 11 mental ill-health to moderately predict LS at age 14, but only among females. In a study focused on coping strategies for MH of 12–13 years-olds children in the United States (Lyon et al., 2014), the authors found that neither internalizing behaviours nor externalizing behaviours predicted LS 6 months later, but LS predicted externalizing behaviours –as well as internalizing behaviours among males, but not among females- 6 months later. Others have studied this question in the transition from late adolescence to adulthood. Using a sample of university students aged 18–22 from China, Bieda et al.(2019) found reciprocal associations over time between LS and a scale of positive MH. Similarly, using a cohort of young people from New Zealand aged 18, 21, 25, 30 and 35, Fergusson et al.(2015) found reciprocal associations between LS and MH (symptoms of internalizing difficulties (depression and anxiety), suicidal ideation, and alcohol and drug use), with fewer MH symptoms being associated with lower LS and increasing LS being associated with more MH symptoms.

The few studies that have explicitly tested for the direction of effects between LS and MH have found evidence suggesting bidirectional effects in the transition from late adolescence to adulthood and no gender differences (i.e. 18–35 years in Fergusson et al., 2015; and 18–22 years in Bieda et al., 2019), and mixed results –and gender differences- in early adolescence (i.e., 12–13 years in Lyon et al., 2014; 11–14 years in Patalay & Fitzsimons,^2018^). To the best of our knowledge, no previous research has studied the direction of effects between LS and MH from middle adolescence to emerging adulthood and how this may differ by gender.

### Transitioning from adolescence to emerging adulthood: a crucial developmental period for mental health and wellbeing

This study examines developmental links between MH and LS in adolescence and emerging adulthood. We focus on the direction of effects between overall LS and MH from age 17 to 21 since this is a period when developmental transitions occur and which is characterized by more vulnerability. The transition from adolescence to emerging adulthood is considered a rough period fraught with many changes in relationships and social roles, and the adoption of new responsibilities (Arnett, 2007; Tanner & Arnett, 2016). Emerging adults’ work and personal lives are characterized by instability (Arnett et al., 2014; Tanner & Arnett, 2016), and this is also a time when young people explore their identity, are in an emotional state of feeling in-between, and change their life goals (Arnett, 2007). Most importantly, emerging adults’ neurodevelopment involves the maturation of the executive and self-regulatory functions and structures of the PFC, which has been implicated in the processing of social-emotional information and the development of common MH disorders such as depression, schizophrenia, and anxiety (Taber-Thomas & Pérez-Edgar, 2015).

Moreover, we study how these developmental links differ between females and males, given that the observational evidence presented above suggested mixed findings regarding the moderating effect of gender in adolescence and emerging adulthood. Thus, our research questions are:

1. What is the direction of effects between MH and LS in the transition from middle adolescence to emerging adulthood?
2. Are there gender differences in these associations?

Considering the evidence from previous research presented above, we hypothesized that (1) LS and MH will predict each other in the transition from mid-adolescence to emerging adulthood, and (2) these developmental links between LS and MH will not differ across gender.

## Methods

### Participants and dataset

Data come from *Understanding Society*: The UK Household Longitudinal Study (UKHLS; University of Essex, Institute for Social and Economic Research, 2021), a longitudinal nationally representative study of UK households (subject to weighting to account for unequal selection and participation probabilities). The study was established in 2009, and all household members aged 16 or older are interviewed annually. We use data from the first ten waves of annual interviews, which took place from 2009 to 2019. At each wave, participants are only enrolled after providing verbal consent to participate. Interviews take place over a 24-months fieldwork period and are computer-assisted. Most interviews are completed in face-to-face mode at the participants’ homes, but a proportion of the sample were interviewed in self-completion mode online since wave 8. For more detailed information about the study design, see Knies (2018).

### Key outcome variables

Our outcome variables of interest are measures of life satisfaction (LS) and mental health (MH). Both are collected as part of the self-completion questionnaire of the UKHLS. LS is measured using a *single item* on a 7-point Likert scale. Participants are asked to choose the number from 0 ‘completely dissatisfied’ to 7 ‘completely satisfied’, which they feel best describes how dissatisfied or satisfied they are with their life as a whole. Higher scores indicate higher LS.

MH is assessed using the short version of the General Health Questionnaire (GHQ-12), a measure designed to capture depressive and anxiety symptoms that is a widely used non-psychotic psychological distress measure with excellent psychometric properties (Goldberg, 1997). Each item (asking about loss of sleep or concentration, among others) has four response categories ranging from 0 ‘not at all’ to 3 ‘much more than usual’. Negatively worded items are reverse-coded. The GHQ-12 scale (dubbed here: MH) sums the 12 items and ranges from 0 ‘least distressed’ to 36 ‘most distressed’. The scale has good reliability at each occasion (age 17 Cronbach’s α = 0.844; age 19 Cronbach’s α = 0.883; age 22 Cronbach’s α = 0.900).

### Sample construction and background characteristics

We constructed six pseudo-cohorts of participating adolescents aged 17 in waves 1 to 6, respectively, who also participated in two later waves when aged 19 and 21 (N = 1,471 unique individuals; C1: 143; C2: 116; C3: 104; C4: 99; C5: 101, C6: 98). Hence, within each cohort, members have different dates of birth but were of the same age at the time of the interview (which took place in different waves of the UKHLS). Subject to applying the appropriate longitudinal sampling weights, our pseudo cohorts’ results are representative of the UK population transitioning from age 17 to 21 in the observation period 2009–2019. Longitudinal weights account for selective attrition and joining of temporary sampling units (TSM) and are routinely supplied with the data (Kaminska & Lynn, 2019). Due to item non-response and individuals with a longitudinal population weight of zero, the sample size is 661 participants (291 males, 370 females) in the final structural equation model.

Regarding the ethnic identity of the sample, the following are applicable. 76.6% of the participants identified as white British/Welsh, Scottish or Northern Irish, whilst the second largest ethnic group was Asian or Asian-British Pakistanis (5.0%). Other major ethnic groups were Asian or Asian-British Indians (4.0%) and Bangladeshis (2.9%), among others. At 17, the majority of the sample (57.5%) attended secondary schools, whilst 25.4% of the sample was attending further or higher education/university institutions. About 17% of the participants at age 17 were not attending any educational institution. The distribution of these characteristics shifted at age 19, when 37.4% of the sample attended university or a higher education institution, 7.2% still studied at secondary schools, and an additional 55.4% were not in education anymore. At age 21, most participants (62.8%) were not attending any educational institutions, whereas a small percentage of participants (35.9%) were attending universities, and a small additional 1.4% were in further or higher education. As expected of a general population adolescent sample, most participants at age 17 (92.4%) reported good or excellent health. This proportion was stable in the two other time points, with 91.1% and 93.7% of the participants reporting good or excellent health at 19 and 21, respectively. Only a small fraction of the sample reported a long-standing illness or impairment on all occasions, with the proportions ranging between 11.2% (age 17) and 13.8% (age 21).

### Theoretical and empirical modelling

As preliminary analyses to ensure construct validity, we first tested the longitudinal measurement invariance of the GHQ-12 to ensure that the measure exhibits good psychometric properties and that it is possible to compare regression coefficients across occasions (Kline, 2016; Liu et al., 2017). Four models of invariance were examined, namely the configural, the metric, the scalar, and the strict (Brown, 2015). The configural model ensures that the latent factor structure of the scale is similar across time points, whereas the metric model tests the hypothesis that the factor loadings of the MH latent factor are equal across occasions (Kline, 2016; Liu et al., 2017). Given the ordered categorical nature of the GHQ-12, the scalar model tests the statistical hypothesis that the items’ thresholds are equal across time points (Liu et al., 2017). Finally, we tested the strict model of invariance, where we imposed equality constraints on the items’ residual variances (Liu et al., 2017). To establish whether a model was invariant, we followed the generally accepted cut-off criterion of a difference in the CFI values between two models equal to/less than 0.010 (Chen, 2007; Cheung & Rensvold, 2002).

**Fig. 1:**
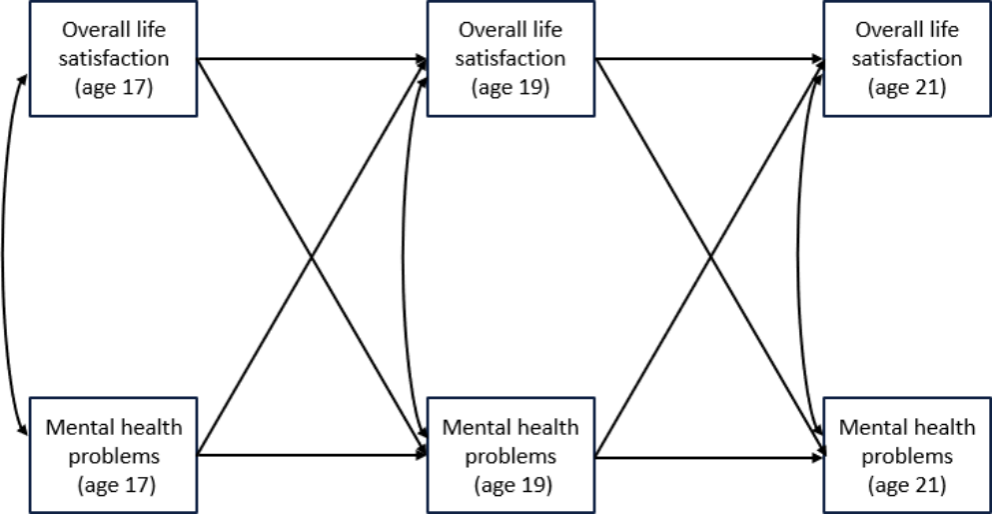
Conceptual model examining the relationship between life satisfaction and mental health problems at ages 17, 19 and 21

Following the preliminary psychometric analyses, a cross-lagged model was estimated using the pooled sample of all pseudo-cohort members (N = 661) and then conducted subgroup comparisons for males (N = 291) and females (N = 370) to test for the potential moderating effects of gender. A cross-lagged panel model was utilized to simultaneously examine the lagged relationship of LS and MH whilst controlling for previous levels of the constructs (Little, 2013). This longitudinal model allowed us also to examine the direction of the relationship (Newsom, 2015) between LS and MH. Figure 1 illustrates the crossed-lagged model structure.

Construction of the pseudo-cohorts, preliminary data management and analyses were carried out using Stata 17 (StataCorp., 2021). Path analysis and measurement invariance were conducted using M*plus* 8.6 (Muthén & Muthén, 2017). All path models were estimated using the maximum likelihood (MLR) estimator, which is robust against non-normality (Yuan & Bentler, 1998), whereas measurement invariance models were estimated using the weighted least squares mean and variance adjusted (WLSMV) estimator to account for the ordered-categorical nature of the item-level data (Muthén & Muthén, 2017). The available longitudinal weight at age 21, as well as stratification and clustering information, were incorporated into the modelling to account for the effects of the complex sampling design of the UKHLS (Kaminska & Lynn, 2019). Fit statistics were used to examine the model fit. A Comparative Fit Index (CFI) value above 0.95 accompanied by Root Mean Square Error of Approximation (RMSEA) and Standardized Root Mean Residual (SRMR) values below 0.06 are considered acceptable (Hu & Bentler, 1999).

## Results

### Preliminary psychometric analyses of the GHQ-12

In the first instance, we tested the configural model, where we specified an equal latent factor structure with auto-correlated residual errors across occasions following the procedure of Liu et al. (2017). All models’ fit indices are presented in Table 1. The configural model displayed an acceptable fit. Having established that the pattern of factor loadings and latent factors was the same across occasions, we specified a model with equality constraints on the factor loadings. This metric model exhibited a good fit according to the fit indices. The difference in CFI values was within the acceptable range. Next, the scalar model with equality constraints on the items’ thresholds resulted in a worse fit than the metric model (|ΔCFI > 0.010), and, thus, a partially scalar model was examined, which was not degraded. Finally, a model with strict invariance was specified and was found to be invariant compared to the partially scalar model. All in all, the GHQ-12 displayed good construct validity and longitudinal measurement invariance across occasions. Thus, we could accurately compare the regression coefficients across timepoints (Byrne, 2012; Liu et al., 2017).

**Table 1:** Goodness of fit indices for measurement invariance models of the GHQ-12

Model	Scaled χ^2^	CFI	|ΔCFI|	TLI	RMSEA	|ΔRMSEA|
Configural	1198.776***	0.955		0.950	0.042	
Metric	1175.157***	0.959	0.004	0.955	0.040	0.002
Scalar	1858.108***	0.916	0.043	0.918	0.053	0.013
Partially Scalar^1^	1281.366***	0.955	0.004	0.955	0.040	0.000
Strict	1281.366***	0.955	0.000	0.955	0.040	0.000

^1^Relaxed constraints on all thresholds of items 10, 11, and two thresholds of item 12

### Testing the direction of effects between overall life satisfaction and mental health problems: a cross-lagged panel analysis

Correlations across core study variables across ages 17, 19, and 21 are shown in Table 2. Overall, there were weak to moderate correlations between LS and MH within and across time.

**Table 2:** Correlations between mental health and life satisfaction at ages 17, 19 and 21, by gender

	LS17	LS19	LS21	MH 17	MH 19	MH 21
LS 17	-	0.321***	0.176**	− 0.454***	− 0.254**	− 0.118*
LS 19	0.245***	-	0.36***	− 0.198***	− 0.405***	− 0.277***
LS 21	0.265***	0.401***	-	− 0.277***	− 0.329***	− 0.455***
MH 17	− 0.457***	− 0.100	− 0.075	-	0.469***	0.395***
MH 19	− 0.144*	− 0.468***	− 0.292***	0.236***	-	0.461***
MH 21	− 0.205**	− 0.312***	− 0.622***	0.222***	0.407***	-
Mean Males (Females)	5.518 (5.478)	5.469 (5.24)	5.315 (5.17)	9.393 (10.942)	9.548 (11.172)	10.021 (12.605)
SD Males (Females)	0.981 (1.184)	0.987 (1.246)	0.966 (1.27)	3.576 (4.452)	3.629 (4.372)	3.784 (5.348)

Note: *** *p < .001;* ** *p < .01*; *p < .05; Correlations for female sample are shown below the diagonal, whereas correlations for the male sample are shown above the diagonal

The results of our first model (Model 1) are reported in Table 3. Model fit indices indicated a satisfactory fit to the data (χ^2^ (4) = 22.720, p = .0001; RMSEA = 0.056 (0.035, 0.080); CFI = 0.956; SRMR = 0.039). In terms of the stability of MH and LS in this transition, the results suggested that age 17 MH was associated with age 19 MH (β = 0.0.337, p = .000), and age 19 MH was associated with age 21 MH (β = 0.0.390, p = .000). The same was true for LS. LS at age 17 was associated with LS at age 19 (β = 0.264, p = .000), and LS at age 19 was associated with overall LS at age 21 (β = 0.308, p = .000). Constraining the stabilities for LS and MH to be the same did not result in worse model fit (age 17 to age 19: Satorra-Bentler Δχ²=3.3718, Δdf = 1, p = .066; age 19 to age 21: Satorra-Bentler Δχ²=2.3896, Δdf = 1, p = .122), suggesting that the stabilities of MH and LS did not significantly differ.

Cross-lagged paths suggested that LS at age 17 was not associated with MH at age 19 (β=-0.042, p = .394) and, conversely, MH at age 17 was not associated with LS at age 19 (β=-0.034, p = .498). However, MH at age 19 years was associated with LS at age 21 years (β=-0.176, p = .000), and LS at age 19 was associated with MH at age 21 years (β=-0.134, p = .008), indicating bidirectional effects.

**Table 3:** Model with standardized regression coefficients indicating associations between mental health and life satisfaction at age 17, 19, and 21 for full sample, for females and for males

	Model 1			Model 2					
	Full sample			Females			Males		
	ß	S.E.	P-Value	ß	S.E.	P-Value	ß	S.E.	P-Value
*Cross-lagged effects*									
Overall LS 17 → MH problems 19	− 0.042	0.049	0.394	− 0.046	0.071	0.516	− 0.051	0.066	0.445
MH problems 17 → Overall LS 19	− 0.034	0.049	0.498	0.016	0.064	0.807	− 0.064	0.080	0.420
Overall LS 19 → MH problems 21	− 0.134**	0.050	0.008	− 0.156*	0.071	0.028	− 0.108	0.060	0.072
MH problems 19 → Overall LS 21	− 0.176***	0.050	0.000	− 0.133	0.069	0.055	− 0.224**	0.075	0.003
*Lagged effects*									
Overall LS 17 → Overall LS 19	0.264***	0.064	0.000	0.252**	0.078	0.001	0.292**	0.105	0.005
Overall LS 19 → Overall LS 21	0.308***	0.057	0.000	0.339***	0.084	0.000	0.271***	0.070	0.000
MH problems 17 → MH Problems 19	0.337***	0.051	0.000	0.215**	0.070	0.002	0.445***	0.075	0.000
MH problems 19 → MH Problems 21	0.390***	0.047	0.000	0.334***	0.062	0.000	0.422***	0.079	0.000
*Within-wave correlations*	r	S.E.	P-value	r	S.E.	P-value	r	S.E.	P-value
LS1- MH1	− 0.454***	0.041	0.000	− 0.458***	0.046	0.000	− 0.456***	0.070	0.000
LS2-MH2	− 0.426***	0.042	0.000	− 0.462***	0.053	0.000	− 0.361***	0.059	0.000
LS3-MH3	− 0.472***	0.040	0.000	− 0.563***	0.046	0.000	− 0.347***	0.067	0.000

Standardized coefficients and confidence intervals. Two-tailed p-values reported

*** p ≤ .001

** p ≤ .01

* p < .05

### Exploring gender differences

To examine gender differences, we estimated a multigroup cross-lagged model (Model 2) (see Table 3). Model fit indices indicate a satisfactory fit to the data for the freely estimated model (χ^2^ (8) = 15.788, p = .0033; RMSEA = 0.060 (0.031, 0.092); CFI = 0.950; SRMR = 0.046). MH at age 17 was associated with age 19 MH among both females (β = 0.215, p = .002) and males (β = 0.445, p = .000). Age 19 MH was associated with age 21 MH among both females (β = 0.334, p = .000) and males (β = 0.422, p = .000). Age 17 LS was associated with age 19 LS among both females (β = 0.252, p = .001) and males (β = 0.292, p = .005), and age 19 LS was associated with age 21 LS both among females (β = 0.339, p = .000) and males (β = 0.271, p = .000). Constraining the stabilities for LS and MH to be the same between age 17 and age 19 did not result in worse fit among either females (Satorra-Bentler Δχ² = 1.8259, Δdf = 1, p = .1766) or males (Satorra-Bentler Δχ²=1.9468, Δdf = 1, p = .1629). The same was true for constraining the stabilities for LS and MH to be the same between age 19 and age 21 (females: Satorra-Bentler Δχ²=0.2265, Δdf = 1, p = .6341; males: Satorra-Bentler Δχ² =2.1932, Δdf = 1, p = .1386). This suggested that the stabilities of MH and LS did not significantly differ for females or males.

Cross-lagged paths demonstrated that MH at age 17 was not associated with age 19 LS among females (β = 0.016, p = .807) or males (β= − 0.064, p = .420). Similarly, age 17 LS was not associated with age 19 MH among females (β=-0.046, p = .516) or males (β= − 0.051, p = .445). On the other hand, when examining cross-lagged paths from age 19 to 21, MH at age 19 was associated with lower levels of LS at age 21 among males (β=-0.224, p = .003) but not among females (β=-0.133, p = .055). However, constraining this path to be equal for females and males did not result in a worse fit (Satorra-Bentler Δχ²=0.6600, Δdf = 1, p = .4166), suggesting that this pathway did not differ for males and females. Similarly, LS at age 19 was associated with MH at age 21 among females (β=-0.156, p = .028) but not males (β=-0.108, p = .072). However, constraining this path to be equal for females and males did not result in a worse fit (Satorra-Bentler Δχ²=0.4765, Δdf = 1, p = .490), suggesting that this pathway did not differ for males and females.

## Discussion

The current study examined associations between LS and MH during the transition from middle adolescence to emerging adulthood. Specifically, we examined the direction of effects between LS and MH from ages 17 to 19 and age 19 to 21 in a pooled sample of six pseudo-cohorts from a powerful household panel study for the United Kingdom, the UKHLS. To the best of our knowledge, this is the first study to have investigated this question in the critical developmental transition from adolescence to emerging adulthood distinguishing between two transition periods (age 17–19 and age 19–21) and exploring gender differences.

Our first research question aimed to study the direction of effects between LS and MH. Initially, we hypothesized reciprocal associations based on evidence drawn from both observational survey studies and neuroscience. Neuroscientific studies suggest that the neural correlates of wellbeing and common MH problems (e.g., depression, anxiety) are similar (King, 2019). Additionally, previous studies have identified a bidirectional association in the transition from late adolescence to adulthood (Bieda et al., 2019; Fergusson et al., 2015) and mixed results in early adolescence (Lyon et al., 2014; Patalay & Fitzsimons, 2018). Notwithstanding the previous evidence, our initial hypothesis was partially rejected: despite the existence of statistically significant within-wave correlations, our findings indicated no longitudinal regressive effects between LS and MH in the age 17–19 transition and bidirectional regressive effects in the age 19–21 transition. Our second research question aimed to examine gender differences in these associations. Given that studies focusing on late adolescent and adult samples found no statistically significant gender differences, we hypothesized that gender does not moderate these structural relationships. The present modelling results partially supported our initial hypothesis. They indicated limited variation between gender groups in the levels of statistical significance of the relationship between LS and MH. Specifically, whereas MH at age 19 predicted lower LS at age 21 in males, LS at age 19 predicted lower MH at age 21 in females.

Taken together, the findings indicate that the association between MH and LS may vary across distinct developmental periods as well as by gender. For example, research using general population samples in the UK shows the existence of reciprocal associations in the 11–14 age transition among females (and unidirectional associations among males, with age 11 LS predicting depressive symptoms at age 14) (Patalay & Fitzsimons, 2018). However, the present study indicated no associations in the 17–19 age transition and reciprocal associations in the age 19–21 transition. This is not surprising as early, middle and late adolescence and emerging adulthood are different stages in terms of the biological and psychosocial changes experienced, with gender differences also being evident (Salmela-Aro, 2011). For example, in countries such as the UK, the age 17–19 period most often implies transitioning from compulsory education to further non-compulsory education or the labour market, whereas the age 19–21 period may imply different educational and/or occupational changes. Other important life domains affected by changes may include relationships, social roles and the adoption of new responsibilities (Arnett, 2007; Tanner & Arnett, 2016), all of which may play an important role in shaping the longitudinal relationship between LS and MH and how this varies across gender. Despite these emerging findings, there remains little literature exploring how the longitudinal associations between LS and MH vary across different developmental periods, and more research is needed. Future research should also explore gender differences with consideration to minority gender identities (e.g., non-binary) whenever possible.

Beyond differences across distinct developmental stages, variation across specific educational and occupational transitions may be another important source of variation across studies. For example, Bieda et al. (2019) found bidirectional associations in a similar age group (age 18–22), but their sample included university students from China transitioning from their first to their last year of undergraduate studies. Unfortunately, due to sample size, we could not examine differences across young people in different educational/occupational transitions, but future research should investigate this. Other sources of variation may include the role of linguistic (Bradshaw, 2015) and cultural differences in how people interpret and respond to wellbeing questions, which have been widely studied in the context of the Western-Eastern divide in wellbeing (Lu & Gilmour, 2015; Leu et al., 2011). Although our findings partly coincide with those of Bieda et al. (2019) from China, more cross-cultural/linguistic research is needed in this area.

Finally, an important source of variation that may explain mixed results in the literature involves differences in how MH and LS are measured in different studies. Our study used a single-item measure of LS, and a multi-item measure of MH (i.e., the GHQ-12 score of psychological distress), whereas -as indicated before- some of these other studies mentioned used other measures of LS and MH, including MH measures that tap into specific MH problems such as depression or anxiety (e.g., Fergusson et al., 2015), measures of coping strategies for MH (Lyon et al., 2014) and measures of positive MH (Bieda et al., 2019). Thus, future research should investigate how the developmental relationship between MH and wellbeing varies when different measures of wellbeing (e.g. eudaimonic wellbeing, affective subjective wellbeing, LS, etc.) and MH (internalizing difficulties, externalizing difficulties, etc.) are considered.

Our study has some valuable strengths due to its longitudinal design and is sufficiently robust with regard to its capacity to reach generalizable conclusions due to its sampling design. However, it also has some limitations. First, research suggests the use of multi-item scales of LS may be preferable to capture individuals’ self-assessment of their life than single-item measures (Diamantopoulos et al., 2012; Casas et al., 2013; Casas et al., 2012). In addition, the study suffered from quite a large degree of non-response attrition in this age group. This was handled with the use of longitudinal weights but remains a limitation that calls for caution when interpreting these results. Furthermore, even though the current configuration of the panel modelling accounts for the autoregressive effects, it does not decompose the effects into interindividual and intraindividual change. Finally, it should be noted that the present data predate the COVID-19 pandemic. We have reason to believe (see Rider et al., 2021; Waite et al., 2021) that the pandemic has exacerbated declines in children’s and adolescents’ wellbeing and increments in MH. However, we do not expect that the pandemic would have changed the substantive psychological meaning of MH and LS, the psychological structure of these constructs, and their relationships in a substantial manner, given the substantial preceding evidence indicating such associations.

In conclusion, the present study contributes to our understanding of the developmental links between MH symptoms and wellbeing in the transition from middle adolescence to adulthood. Specifically, the findings illustrate that the magnitude and the statistical significance of the association between wellbeing and MH fluctuate substantially depending on the age of the participants. Furthermore, it was shown that gender is not a considerable moderator of this relationship since gender differences were not profound in the transition under study.

## Data Availability

The data are freely available through the UK Data Archive (https://www.data-archive.ac.uk/find/).
